# Association of *ABCA4* Gene Polymorphisms with Cleft Lip with or without Cleft Palate in the Polish Population

**DOI:** 10.3390/ijerph182111483

**Published:** 2021-10-31

**Authors:** Alicja Zawiślak, Krzysztof Woźniak, Xabier Agirre, Satish Gupta, Beata Kawala, Anna Znamirowska-Bajowska, Katarzyna Grocholewicz, Jan Lubiński, Felipe Prosper, Anna Jakubowska

**Affiliations:** 1 Department of Interdisciplinary Dentistry, Pomeranian Medical University, 70-111 Szczecin, Poland; katarzyna.grocholewicz@pum.edu.pl; 2 Centro de Investigación Médica Aplicada, IDISNA, Universidad de Navarra, Avenida Pío XII-55, 31008 Pamplona, Spain; xaguirre@unav.es (X.A.); fprosper@unav.es (F.P.); 3 Department of Maxillofacial Orthopaedics and Orthodontics, Institute of Mother and Child, 01-211 Warsaw, Poland; 4 Department of Orthodontics, Pomeranian Medical University, 70-111 Szczecin, Poland; krzysztof.wozniak@pum.edu.pl; 5 Hereditary Cancer Center, Department of Genetics and Pathology, Pomeranian Medical University, 70-111 Szczecin, Poland; satish1482@gmail.com (S.G.); lubinski@pum.edu.pl (J.L.); anna.jakubowska@pum.edu.pl (A.J.); 6 Department of Dentofacial Orthopaedics and Orthodontics, Wrocław Medical University, 50-425 Wrocław, Poland; ortodoncja@umed.wroc.pl (B.K.); aznamirowska.b@gmail.com (A.Z.-B.)

**Keywords:** congenital condition, cleft lip, cleft palate, genetic variation, single nucleotide polymorphism, *ABCA4*

## Abstract

Background: Non-syndromic cleft lip with/without cleft palate (NSCL/P) is a common congenital condition with a complex aetiology reflecting multiple genetic and environmental factors. Single nucleotide polymorphisms (SNPs) in *ABCA4* have been associated with NSCL/P in several studies, although there are some inconsistent results. This study aimed to evaluate whether two SNPs in *ABCA4*, namely rs4147811 and rs560426, are associated with NSCL/P occurrence in the Polish population. Methods: The study included 627 participants: 209 paediatric patients with NSCL/P and 418 healthy newborn controls. DNA was isolated from the saliva of NSCL/P patients and from umbilical cord blood in the controls. Genotyping of rs4147811 and rs560426 was performed using quantitative PCR. Results: The rs4147811 (AG genotype) SNP in *ABCA4* was associated with a decreased risk of NSCL/P (odds ratio (OR) 0.57; 95% confidence interval (CI) 0.39–0.84; *p* = 0.004), whereas the rs560426 (GG genotype) SNP was associated with an increased risk of NSCL/P (OR 2.13; 95% CI 1.31–3.48; *p* = 0.002). Limitations: This study—based on the correlation between single genetic variants and the occurrence of different phenotypes—might have limited power in detecting relevant, complex inheritance patterns. ORs are often low to moderate when investigating the association of single genes with the risk of a complex trait. Another limitation was the small number of available NSCL/P samples. Conclusions: The results suggest that genetic variations in *ABCA4* are important risk markers of NSCL/P in the Polish population. Further investigation in a larger study group is warranted.

## 1. Introduction

Non-syndromic cleft lip with or without cleft palate (NSCL/P) is a common complex orofacial congenital condition occurring in 1 in 700 births [1] and affecting 135,000 newborns worldwide [2]. The incidence of NSCL/P varies between ethnic groups and geographic regions, with high rates reported in parts of Latin America and Asia and low rates reported in Israel, South Africa, and Southern Europe. High rates of isolated cleft palate are also reported in Canada and parts of Northern Europe, and low rates are reported in parts of Latin America and South Africa [3]. The cleft palate only (CPO) phenotype is less common, with a prevalence of about 1–25 in 10,000 births in most ethnic groups [4].

Clinically, NSCL/P influences many aspects of everyday life (e.g., eating, speech, and facial appearance) and can significantly increase the overall healthcare needs of affected individuals. Treatment of children with this malformation requires an interdisciplinary approach. Medical care usually lasts from birth to adulthood and always requires the care of an orthodontist, dentist, paediatrician, phoniatrist, speech therapist, and maxillofacial surgeon. A large number of paediatric patients who suffer from congenital malformations represent a challenge for the public dental healthcare system. [5]. The aetiology of NSCL/P is complex, involving both genetic and environmental factors [6]. Indeed, several studies indicate that there is a strong genetic component to this congenital disability. For example, the risk of NSCL/P is tripled in siblings compared with the general population, and the risk is estimated at 25–45% for monozygotic twins compared with 3–6% for heterozygotic twins. Moreover, the risk of orofacial malformations for first-degree relatives is estimated at 4%; for second-degree relatives, it is 0.67%; and for third-degree relatives, it is 0.3% [7]. Nonetheless, the lack of total coincidence of orofacial clefts in monozygotic twins indicates that exogenous factors also play important roles in the aetiology of NSCL/P [8].

Although enormous progress has been made in understanding the genetic aetiology of syndromic orofacial clefting [9], the identification of specific genetic variants involved in NSCL/P has progressed at a slower pace, primarily due to its multifactorial nature [1]. Some studies have shown that several genes and loci are associated with oral clefts [10,11,12]. More recently, however, advances in methodologies have accelerated the discovery of loci conferring susceptibility to isolated NSCL/P through the use of genome-wide association studies (GWAS). For example, the chromosomal locus 8q24 was found to be associated with NSCL/P in a Central European population [13] as well as in a paediatric cohort of European descent recruited from the Greater Philadelphia area [14], a Brazilian population [15], and a population with European ancestry [16]. Meanwhile, NSCL/P was associated with the 1p22 (*ABCA4* gene) and 20q12 (*MAFB* gene) loci in an Asian population [16]. Finally, the 17q22 and 10q25 (*VAX1* gene) loci were found to be associated with NSCL/P in patients of European origin recruited in the United States [17].

The *ABCA4* gene encodes a membrane-associated protein that is a member of a superfamily of ATP-binding cassette (ABC) transporters that transport various molecules across extra- and intracellular membranes [18]. GWAS results have revealed an association between missense mutations within *ABCA4* and a spectrum of retinal disorders in individuals with NSCL/P [16,19]. Therefore, in this study, we sought to evaluate the association of two Single Nucleotide Polymorphisms (SNPs) located in *ABCA4* (1p22), namely rs560426 and rs4147811, with the occurrence of NSCL/P in a Polish population. To our knowledge, SNPs of this gene have not yet been studied in the Polish population.

## 2. Subjects and Methods

### 2.1. Study Population

The study group comprised unselected paediatric patients with NSCL/P and healthy controls. All patients included in the study were under orthodontic treatment, either in the Department of Orthodontics at the Pomeranian Medical University in Szczecin or in the Department of Dentofacial Orthopedics and Orthodontics at the Wrocław Medical University.

In the group of 418 NSCL/P patients, the clinical diagnostics of existing congenital defects and differential diagnostics for monogenic syndromes related to NSCL/P were based on medical history and physical examination. The type of cleft was assessed according to World Health Organisation classification (International Statistical Classification of Diseases and Related Health Problems—ICD 10; Congenital malformations, deformations and chromosomal abnormalities section Q35–Q37) [20].

The control group consisted of 418 anonymous children (average age 14.0 ± 10.2 years) whose genetic material was obtained from umbilical cord blood and stored in the biobank of the Department of Genetics and Pathology at the Pomeranian Medical University in Szczecin. The controls were age and geographically matched to the NSCL/P patients. In total, 180 children from Szczecin and 238 from Wrocław and Opole (79 km from Wrocław) were included.

### 2.2. Ethical Approval

The study was approved by the Bioethics Committee of the Pomeranian Medical University in Szczecin. The patients, their parents, or their legal guardians were informed about the purpose of the study and gave their informed written consent.

### 2.3. Sample Preparation

In the NSCL/P group, 2 mL samples of saliva were collected from each participant using collective Oragene kits (DNA Genotek Inc., Ottawa, ON, Canada). The participants were asked not to consume any solid food for 30 min prior to biological material collection. The samples were kept dry and protected from light at room temperature. DNA was isolated using automatic Chemagen sets. The DNA extracted from the samples was preserved in a freezer at −20 °C. In the control group, umbilical cord blood DNA was extracted using the method described by Lahiri et al. [21].

### 2.4. Genotyping

Genotyping of rs4147811 and rs560426 was performed using the real-time PCR-based TaqMan technique with LightCycler 480 II (Roche Diagnostics, Basel, Switzerland). The mixture (5 μL total) contained 2.5 μL of the LightCycler 480 Probes Master Mix (Roche Diagnostics), 0.0625 μL of each SNP TaqMan Genotyping Assay × 40 (Applied Biosystems, Waltham, MA, USA), 1.4375 μL of deionised water (Roche Diagnostics), and 1 μL of DNA (25 ng/μL). The PCR conditions are presented in Table 1. On each plate, four negative controls without DNA were included to monitor contamination.

### 2.5. Statistical Analysis

To assess the risk of orofacial cleft occurrence, an odds ratio (OR) was calculated with a 95% confidence interval (CI) using logistic regression. The most common genotype was used as a reference. The cleft risk was assessed for each genotype. The significance of particular logistic regression rates was assessed using the Wald’s test. Statistica 10.0 (StatSoft, Tulsa, OK, USA) and R 3.0.2 (The R Foundation for Statistical Computing) were used for statistical analysis. *p*-values of less than 0.05 were considered significant.

## 3. Results

The study group comprised 209 patients with NSCL/P and 418 healthy controls (age range, 4–30 years; average age 17.4 ± 13.6 years). All of the patients’ relatives within two generations were Polish.

The NSCL/P group consisted of 91 women (43.5%) and 118 men (56.5%). In 113 cases (54.1%), unilateral cleft of the lip and the hard palate was observed; in 45 cases (21.5%), bilateral cleft of the lip and hard palate was observed; in 32 cases (15.3%), CPO was observed; and in 19 cases (9.1%), isolated cleft lip was observed. The characteristics of the NSCL/P patients are presented in Table 2.

As shown in Table 3, a logistic regression analysis demonstrated that the rs4147811 SNP was related to a decreased risk of NSCL/P. In particular, the risk (OR) of NSCL/P was significantly decreased in those with the AG (*p* = 0.004) and AA + AG genotypes (*p* = 0.005) but not the AA genotype (*p* = 0.144). Meanwhile, the rs560426 SNP was related to an increased risk (OR) of orofacial cleft (Table 4). The GG (*p* = 0.002) and AG + GG (*p* = 0.036) genotypes were associated with an increased risk of NSCL/P; however, the AG genotype was not significantly related (*p* = 0.229).

## 4. Discussion

In this study, we confirmed the influence of two SNPs (rs4147811 and rs560426) within *ABCA4* on the risk of NSCL/P in a Polish population. Although there is much (albeit conflicting) evidence on the relationship between rs560426 and cleft palate, the role of rs4147811 in the formation of this congenital abnormality remains unclear. We found that rs4147811 is associated with a decreased risk of NSCL/P whereas rs560426 is associated with an increased risk of NSCL/P.

GWAS conducted in European and Asian populations involving case-parent trios [16] revealed that two novel genes were associated with NSCL/P: *MAFB* and *ABCA4.* In particular, rs13041247 of *MAFB* (the most significant SNP) had an OR (per minor allele) of 0.704 (95% CI 0.635–0.778, *p* = 1.44 × 10^−11^) and rs560426 of *ABCA4* (the most significant SNP) had an OR (per minor allele) of 1.432 (95% CI 1.292–1.587, *p* = 5.01 × 10^−12^), which is similar to our results. In the same study, the investigators identified two previously identified regions (8q24 and *IRF6*) with genome-wide significance. Upon stratifying trios into European and Asian ancestry groups, Beaty et al. [16] found that the estimated effect sizes remained similar, although the differences were statistically significant. These findings from replication studies of families of European and Asian ancestries provide strong evidence for the association of NSCL/P with 8q24 (in European families) and with *MAFB* and *ABCA4* (in Asian families) [16].

In contrast to our findings, Pan et al. [22] found no associations between rs560426 and NSCL/P susceptibility. In their study, 396 NSCL/P and 384 healthy subjects were included to evaluate the risk of NSCL/P and their subgroups in a Chinese Han population. The investigators found that the overall genotype and allele frequencies for rs13041247 were significantly different between NSCL/P and healthy subjects but not for rs560426. A further stratified analysis identified the apparent protection against cleft lip with or without cleft palate, cleft lip with cleft palate, and cleft lip only with rs13041247. However, none of the rs560426 genotypes or alleles seemed to be associated with the overall risk of NSCL/P or its subgroups.

Leslie et al. [23] demonstrated a clear craniofacial expression of the *ARHGAP29* gene and identified variants that were collectively overrepresented in cases with NSCL/P compared with an unaffected control group. According to them, *ARHGAP29* is a more plausible candidate for cleft lip and palate than *ABCA4*. Although these two genes are not located within the same linkage disequilibrium (LD) block, the investigators hypothesise that significant genome-wide associations might be partly driven or determined by multiple rare variants, which could be located in adjacent LD blocks [24]. Leslie et al. [23] recognised rs560426 as a surrogate for the locus and believed that rs560426 is in an LD with other common aetiologic SNPs, acting upon very distant targets. Such hypotheses highlight the importance of examining these related regions more widely.

In the study by Leslie et al. [23], 180 NSCL/P and healthy subjects from the US and the Philippines were screened for mutations in *ARHGAP29* protein-coding exons. The recognised nine exons with variations in *ARHGAP29* were then screened in an independent set of 872 NSCL/P and 802 control subjects. During the *ARHGAP29* sequencing, eight potentially deleterious variants were identified, including a frameshift mutation and one missense variant. As further evidence, *ARHGAP29* showed craniofacial expression and reduced levels in an *IRF6*-deficient mouse (*IRF6* plays a critical role in craniofacial development). Moreover, Savastano et al. [25] obtained DNA samples from 173 families in Brazil and 15 in the UK (with an average number of affected subjects of 2.6/family) and identified 10 rare *ARHGAP29* variants (5 missense, 1 in-frame deletion, and 4 loss-of-function; the last ones seemed to contribute to NSCL/P). Savastano et al. also performed genotyping of rs560426 and rs4147811 (1p22), and rs642961 (*IRF6*). However, the authors concluded that both SNPs at 1p22 as well as rs642961 at *IRF6* do not significantly contribute to the penetrance and do not support an interaction between *ARHGAP29* and *IRF6*.

In another study, Yuan et al. [26] found evidence for the association of rs560426 with orofacial cleft in a Hispanic population in the US. In particular, they aimed to determine whether rs560426 and rs481931 in *ABCA4*, and rs13041247 and rs11696257 in *MAFB,* were associated with NSCL/P in a dataset comprising 182 NSCL/P families with a positive family history (121 non-Hispanic and 60 Hispanic) and 464 case-parent duos/trios with a negative family history (294 non-Hispanic and 170 Hispanic). For *ABCA4*, only rs481931 (but not rs560426) showed a significant association with NSCL/P in non-Hispanic families, whereas both rs481931 and rs560426 were associated with NSCL/P (*p* < 0.05) in the Hispanic dataset. After stratification by family history, the negative family history subset was responsible for most of the association in the non-Hispanic dataset. Meanwhile, there was evidence for an association with rs560426 in the negative family history subset in the Hispanic dataset and for rs481931 in the positive family history subset. Minor associations were detected for rs11696257 and rs13041247 in the Hispanic dataset, whereas no association was found for the non-Hispanic dataset. In contrast, Beaty et al. [16] found no relationship between NSCL/P and *ABCA4* SNPs in a South and Central American population, perhaps due to population (ethnic) differences.

Fontoura et al. [27] also found evidence of an association between SNPs in *ABCA4* and NSCL/P. In their case–control study in a Brazilian population. The investigators evaluated whether rs13041247 and rs11696257 in *MAFB*, and rs560426 and rs481931 in *ABCA4* were associated with NSCL/P. They genotyped 812 Caucasian individuals (400 NSCL/P and 412 healthy subjects) and compared the allele frequencies for NSCL/P (and its subgroups) and control subjects. They found a strong association of rs560426 with NSCL/P, unilateral and right NSCL/P, and bilateral NSCL/P and a borderline association of rs481931 with NSCL/P and bilateral NSCL/P; however, there was no association with any of the *MAFB* SNPs.

In contrast to our findings, neither rs560426 nor rs4147811 in *ABCA4* was associated with orofacial cleft in a Nigerian population [28]. In their study, Butali et al. [28] collected and investigated 118 cases (76 triads and 42 dyads) with a family history of clefts (69 cases with NSCL/P, 38 with cleft lip, and 11 with CPO). However, the family cases were obtained from the mothers as part of their family history of cleft information based on questionnaires, and no physical examination was carried out on the relatives with clefts to ascertain the cleft type. For a case–control comparison, the investigators recruited two controls per case, matched by geopolitical region, birth month, and gender. A total of 166 control samples (164 dyads and two triads) were recruited from individuals at hospitals in each geopolitical zone. The study concentrated on the genes *MSX1*, *IRF6*, *FOXE1*, *FGFR1*, *FGFR2*, *BMP4*, *MAFB*, *ABCA4*, *PAX7*, and *VAX1* and on the chromosome 8q region. A missense mutation A34G in *MSX1* was observed in nine cases and four HapMap controls. However, no other associations were identified, including gene variants in *ABCA4*.

As far as the Polish population is concerned, three studies have been conducted by Mostowska et al. [29,30,31]. In 2010, Mostowska et al. [29] analysed 18 polymorphisms of *FOXE1*, *IRF6*, *MSX1*, *PAX9*, *TBX10*, *FGF10*, *FGFR1*, *TGFa*, *TGFb3*, *SUMO1*, and the chromosomal region 8q24 in a group of 175 NSCL/P subjects and a matched control group. Strongly significant associations with NSCL/P risk were observed for rs642961 in *IRF6* (OR (AG + AA vs. GG) = 1.635, 95% CI 1.153–2.319, *p* = 0.005) and rs987525 in 8q24 (OR (AC + AA vs. CC) = 1.962, 95% CI 1.382–2.785, *p* = 1.4 × 10^−4^). The investigators also identified a borderline association of rs2350350 in the *SUMO1* locus with an increased risk of NSCL/P (OR (CG vs. GG) = 1.580; 95% CI 1.056–2.363, *p* = 0.025).

In another study, Mostowska et al. [30] investigated 24 SNPs of 11 stress-related genes (*COMT*, *CRHR1*, *FKBP5*, *GABRA6*, *HSD11b2*, *MAOA*, *NPY*, *NR3C1*, *SERPINA6*, *SLC6A4*, and *TPH2*) in a group of 220 healthy mothers of children with NSCL/P and a matched control group of 210 individuals. They found—using restriction fragment-length polymorphism and high-resolution melting analysis—that polymorphisms in *SLC6A4*, *TPH2*, and *SERPINA6* seem to be maternal factors that increase the risk of having a child with NSCL/P. Associations with NSCL/P were found for rs2020942 in *SLC6A4* and rs10879357 in *TPH2* (OR = 1.720, 95% CI 1.158–2.553, *p* = 0.0069; and OR = 1.837, 95% CI 1.226–2.753, *p* = 0.003, respectively).

Mostowska et al. [31] also studied variants located at chromosomal regions 1p22.1, 10q25.3, 17q22, and 20q12 in a group of 206 NSCL/P subjects and a matched control group of 446 individuals. Using a recessive model, both rs7078160 and rs4752028 (polymorphisms located at 10q25.3) were associated with a greater than four-fold increase of NSCL/P risk (OR = 4.536, 95% CI 1.678–12.265, *p* = 0.0012 and OR = 4.573, 95% CI 1.817–11.512, *p* = 0.0004, respectively). Additionally, rs227731 (located at 17q22) increased the NSCL/P risk when analysed under a dominant model (OR = 1.732, 95% CI 0.184–2.253, *p* = 0.0044). There was also a borderline association for the 1p22.1 locus (rs481931). The OR for rs560426 was 0.855, but unlike our findings, it did not achieve statistical significance. This lack of a significant association for rs560426 in the study by Mostowska et al. [31] could reflect differences in the geographical origins of the cases compared with those in our study.

## 5. Limitations

Many of the studies involving NSCL/P have yielded inconsistent results. We are aware that a study design based on the correlation between single genetic variants and the occurrence of different phenotypes might have limited power in detecting relevant complex inheritance patterns (e.g., synergic involvement of different SNPs), and further study is needed to confirm the reported associations. Indeed, ORs are almost always low to moderate when investigating the association of single genes with the risk of a complex trait that is likely to be governed by a considerable number of genes. This reflects the fact that a specific phenotype results from a combination of different genes (each of which contributes only to a small effect) and environmental factors. Therefore, it would be appropriate to consider the gene–environment interactions to explain the remaining genetic risk of the non-syndromic forms of orofacial clefts. Another limitation is the small number of available NSCL/P samples, and further investigation should be conducted in a larger cohort.

## 6. Conclusions

This is the first study to investigate the association between *ABCA4* genotypes and NSCL/P in the Polish population. Our findings suggest that rs4147811 is associated with a decreased risk of NSCL/P, whereas rs560426 is associated with an increased risk of NSCL/P. Overall, this study contributes to our understanding of genetic factors related to NSCL/P; however, further investigation in a larger population is required.

## Figures and Tables

**Table 1:** PCR reaction parameters with TaqMan probes.

Program	Cycle	Time	Temperature (°C)	Number of Cycles
Incubation	HotStart	10 min	95	1
Amplification and detection	Denaturation	10 s	95	40–45
	Primer annealing	30 s	60	
	Extension	1 s	72	
Cooling	Cooling	30 s	40	
Colour compensation	Denaturation	1 s	95	
	Cooling	30 s	40	
	Hybridization		67	1 reading/°C
	Cooling	40 s	40	
Melting curve	Hybridization	1 s	60	
	Hybridization		61	5 readings/°C
Cooling	Cooling	40 s	40	

**Table 2:** Characteristics of patients with NSCL/P.

		Number and Proportion of Individuals
Gender	Female	91 (43.5%)
	Male	118 (56.5%)
Type of cleft	Unilateral cleft of the lip and the hard palate	113 (54.1%)
	Bilateral cleft of the lip and the hard palate	45 (21.5%)
	Cleft palate only	32 (15.3%)
	Isolated cleft lip	19 (9.1%)

**Table 3:** NSCL/P risk and rs4147811 polymorphism in *ABCA4*.

Genotype	OR	95% CI	*p*
GG	Ref.		
AA	0.67	0.39–1.15	0.144
AG	0.57	0.39–0.84	0.004
AA + AG	0.59	0.41–0.85	0.005

**Table 4:** NSCL/P risk and rs560426 polymorphism in *ABCA4*.

Genotype	OR	95% CI	*p*
AA	Ref.		
AG	1.29	0.85–1.96	0.229
GG	2.13	1.31–3.48	0.002
AG + GG	1.52	1.03–2.24	0.036

## Data Availability

All data are available from the corresponding author upon request.
